# Quercetin and Vitamin C: An Experimental, Synergistic Therapy for the Prevention and Treatment of SARS-CoV-2 Related Disease (COVID-19)

**DOI:** 10.3389/fimmu.2020.01451

**Published:** 2020-06-19

**Authors:** Ruben Manuel Luciano Colunga Biancatelli, Max Berrill, John D. Catravas, Paul E. Marik

**Affiliations:** ^1^Division of Pulmonary and Critical Care Medicine, Eastern Virginia Medical School, Norfolk, VA, United States; ^2^Frank Reidy Research Center for Bioelectrics, Old Dominion University, Norfolk, VA, United States; ^3^Policlinico Umberto I, La Sapienza University of Rome, Rome, Italy; ^4^Department of Respiratory Medicine, St. Peter's Hospital, Surrey, United Kingdom; ^5^School of Medical Diagnostic & Translational Sciences, College of Health Sciences, Old Dominion University, Norfolk, VA, United States

**Keywords:** SARS-Cov-2, COVID-19, vitamin C, quercetin, flavonoids, antiviral, Coronavirus, immunonutrition

## Abstract

Severe Acute Respiratory Syndrome Coronavirus-2 (SARS-CoV-2) represents an emergent global threat which is straining worldwide healthcare capacity. As of May 27th, the disease caused by SARS-CoV-2 (COVID-19) has resulted in more than 340,000 deaths worldwide, with 100,000 deaths in the US alone. It is imperative to study and develop pharmacological treatments suitable for the prevention and treatment of COVID-19. Ascorbic acid is a crucial vitamin necessary for the correct functioning of the immune system. It plays a role in stress response and has shown promising results when administered to the critically ill. Quercetin is a well-known flavonoid whose antiviral properties have been investigated in numerous studies. There is evidence that vitamin C and quercetin co-administration exerts a synergistic antiviral action due to overlapping antiviral and immunomodulatory properties and the capacity of ascorbate to recycle quercetin, increasing its efficacy. Safe, cheap interventions which have a sound biological rationale should be prioritized for experimental use in the current context of a global health pandemic. We present the current evidence for the use of vitamin C and quercetin both for prophylaxis in high-risk populations and for the treatment of COVID-19 patients as an adjunct to promising pharmacological agents such as Remdesivir or convalescent plasma.

## Introduction

It is serendipitous (or perhaps indicative of hard work) that the Nobel prize winner Szent-Gyorgyi discovered both ascorbic acid (vitamin C) and the flavonoid quercetin (at the time labeled vitamin P) (1). Ascorbic acid is an essential vitamin with known antiviral properties (2) which is under investigation for its beneficial effects during the stress response in sepsis and critically ill patients (3).

Vitamin C exerts its antiviral properties by supporting lymphocyte activity, increasing interferon-α production, modulating cytokines, reducing inflammation, improving endothelial dysfunction, and restoring mitochondrial function (4–6). There are also suggestions that vitamin C may be directly viricidal (7). These *in vitro* effects, as we previously discussed (2), constitute a reflection of both the supra-physiological concentrations of ascorbate and the interaction between vitamin C and metal-containing culture media—both of which are pro-oxidant, generating reactive oxygen species.

Quercetin (also known as 3,3′,4′5,7-pentahydroxyflavone) is a widely distributed plant flavonoid, found in several vegetables, leaves, seeds, and grains, where it is conjugated with residual sugars to form quercetin glycosides (8). Studies suggest that quercetin supplementation may promote antioxidant (9), anti-inflammatory, antiviral (10), and immunoprotective effects (11). Quercetin has been studied in various types and models of viral infection due to its promising antiviral effects in inhibiting polymerases (12), proteases (13), reverse transcriptase (14), suppressing DNA gyrase, and binding viral capsid proteins (15, 16).

In this review we collate the evidence of the antiviral properties of quercetin, describe its biologic action and pharmacokinetics profile, expand on our previous review of vitamin C, discuss their synergistic actions, and propose this experimental multi-drug approach for the prevention and treatment of SARS-CoV-2/COVID-19 pandemic.

## Chemistry of Quercetin

**Figure 1:**
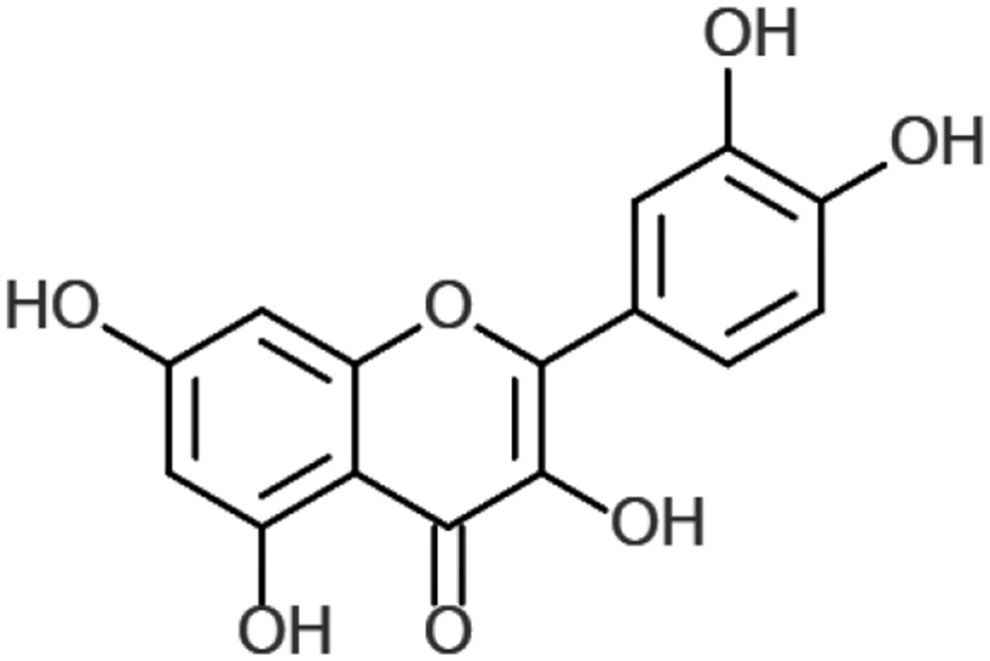
Chemical structure of quercetin. Created with ChemDoodle Web with permission (18).

In plants, quercetin is produced from the phenylpropanoid pathway and is ultimately derived from phenylalanine. It is converted to 4-coumaroyl-CoA, via phenylalanine ammonia-lysate, to cinnamate-4-hydroxylase and 4-coumaroyl-CoA-ligase. This is combined with malonyl-CoA in a 1:3 ratio by 7,2′-dihydroxy-4′methoxyisoflavanol synthase to form tetrahydroxy chalcone. This in turn is converted to naringenin and to eriodyctiol through flavonoid 3′-hydroxylase. Finally, eriodyctiol is hydroxylated and converted to quercetin (Figure 1) using flavanol synthase (17).

## Biology of Quercetin

Flavonoid compounds, such as quercetin, were initially studied for their biological activity in affecting capillary wall resistance (19) and continue to be investigated for their effects on vascular tension (20). Dietary supplements differ, but often contain the free form of quercetin—quercetin aglycone—under the FDA national drug code numbers 65448-3085, 65448-3005 (21). Once consumed, quercetin passes predominantly unaltered into the large intestine (22). Quercetin acts as a free radical scavenger, donating two electrons via o-quinone/quinone methide (23); both *in vitro* and *in vivo* (24, 25) studies implicate quercetin as a potent antioxidant. This antioxidant activity may also be potentiated by vitamin C (26), as will be discussed below. There is also significant longstanding interest in the anti-inflammatory activity of quercetin, as it has been suggested to be a key mediator in the cardiovascular protective element of the “Mediterranean” diet (27). This biological rationale is secondary to quercetin's free radical scavenging capacity, alongside diverse roles identified in *in vitro* and *in vivo models* including: inhibition of platelet aggregation (28), inhibition of lipid peroxidation (29), and its inhibitory effects on pro-inflammatory mediators such as lipoxygenase (30) and phospholipase A2 (31). This anti-inflammatory effect is primarily mediated by flavonoid activity on arachidonic acid metabolism and the associated leukotriene/prostaglandin pathways. Furthermore, 3-methyl-quercetin, a quercetin metabolite, displays stimulatory effects on nasal epithelial cell ciliary beat frequency, both *in vitro* and *in vivo*, when administered either alone or with absorption enhancer HP-β-CD (32). Quercetin also affects the function of several lipids, protein tyrosine, and serine/threonine kinases (33, 34), such as phosphatidylinositol (PI)-3-kinase and inducible nitric oxide synthase (NOS2) (35, 36).

## Beneficial Effects of Vitamin C and Quercetin in Viral Infections

There is a tremendous amount of literature supporting the antiviral properties of quercetin, in both *in vitro* and *in vivo* experiments. Quercetin inhibits several respiratory viruses in cultured cells (16, 37). It inhibits the cytopathic effects provoked by many serotypes of rhinovirus, echovirus (type 7, 11, 12, and 19), coxsackievirus (A21 and B1), and poliovirus (type 1 Sabin) at a minimal inhibitory concentration of 0.03 to 0.5 μg/ml in Hela or WI-38 cells (38). Quercetin also significantly reduces plaque formation by RNA and DNA viruses [Respiratory Syncytial Virus (RSV), Polio type 1, parainfluenza type 3, and Herpes Simplex Virus-1(HSV-1)] displaying anti-infective and anti-replicative properties (39). It inhibits the replication of cytomegalovirus (CMV) inoculated HeLa cells at a half inhibitory concentration (IC50) of 3.2 ± 0.8 μM and with a selectivity index (SI) of 22 (40). Dengue virus type 2 (DENV-2) replication in Vero cells is inhibited by quercetin at an IC50 of 35.7 μg/mL, causing a DENV-2 RNA reduction of 67%. This is attributed to quercetin's ability to either block virus entry or inhibit viral replication enzymes such as viral polymerases (41).

*In vivo* studies indicate that mice inoculated with meningoencephalitis virus are protected from lethal infection by quercetin (30 or 40 mg/Kg BID, po, for 4 days) in a dose dependent manner (42). These beneficial effects are abolished if the compound is administered for <3 days, once per day or via subcutaneous injection. This may suggest that the antiviral effects may be dependent on a minimum inhibitory concentration or from some form of metabolic drug conversion (42). Quercetin treatment also displayed a beneficial effect in immunocompetent mice infected with Mengo virus, where it lessened the severity of organ damage (43). Athletes supplemented with quercetin are protected from stress-induced susceptibility to upper respiratory tract infection (44)—which was not related to immunomodulation (45, 46).

Vitamin C is an essential nutrient involved in a diverse array of immune functions; its supplementation has demonstrated beneficial effects in different types of viral infections. Reduced levels of ascorbate have been found in patients with viral infections (47), sepsis (48), sepsis-related ARDS (49), and other critical illness (50). During infection, vitamin C is necessary for neutrophil killing (51), is concentrated within macrophages (52), is responsible of T cell maturation (53), and promotes phagocytosis and apoptosis of spent neutrophils (4). It is not surprising, therefore, that viral infections, depending on their severity, are associated with an increased metabolism and reduced circulating ascorbate.

Vitamin C has improved survival in different murine models of lethal infection. Mice infected with Venezuelan encephalitis virus and treated with vitamin C (50 mg/kg) exhibit half the mortality of controls with associated reductions in viral titers, lipid peroxidation products, and NO content (54). Mice incapable of synthetizing vitamin C (*L*-Gulono-gamma-lactone oxidase nulls) were infected with influenza; mice not receiving supplemental vitamin C exhibited greater lung pathology scores despite no differences in viral titers (55). In restraint-stressed mice with H1N1 viral-induced pneumonia, vitamin C reduced mortality dose-dependently (100% vs. 80% vs. 50% at 0, 125, and 250 mg/kg/day) and reduced capillary-alveolar structural damage (56). Mice inoculated with Rabies+ mouse brain cells and treated with daily 100 mg/kg IM vitamin C exhibited nearly half the mortality of controls (57).

The only human study of vitamin C has been in USSR soldiers with severe viral infection indicated vitamin C supplementation (300 mg/day) protected from influenza-associated pneumonia and was associated with shorter hospital stays (58).

Vitamin C administration (i.v. 5 g/day twice/week) in patients with herpes zoster exhibited a lower incidence of postherpetic neuralgia (31.1% vs. 57.1%) and at study end (week 16) there was a lower pain score in the treatment group (0.64+/−0.9 vs. 1.98 +/−0.7) (59). Vitamin C administered at 1 g BID to 133 patients, reduced the risk (OR 0.25) of herpes simplex keratitis (HSK) recurrence (60), in accordance with previous studies indicating reduced ascorbate availability in the eye (61). It is noteworthy that a growing number of case reports of virus-related acute respiratory distress syndromes (ARDS) indicate successful treatment with intravenous high doses of Vitamin C (62, 63).

Co-administration of quercetin (12.5 mg/kg/week) and vitamin C and B_3_ in a murine model of exercise-induced susceptibility to influenza H1N1 prolonged time-to-death (median time to death: placebo 9.0 ± 0.33 vs. quercetin 16.5 ± 1.2) and improved survival (mortality: placebo 74% vs. quercetin 52%) when compared to mice receiving only vitamins B3 and C (64). An older, small clinical trial identified the combination of flavonoids and ascorbic acid (1:1 ratio) as beneficial for respiratory infection (200 mg TID) (65).

### Inhibiting Virus Entry

Cell entry is a crucial step during viral infection and has been studied as a potential target of antiviral treatments (66–68). In an *in vitro* model of H1N1 and H3N2 influenza infection of MDCK cells, quercetin demonstrated reduced cytopathic effect 48 h post-infection (69). This effect was observed when quercetin was administered during viral entry (0–2 h), was maximal with quercetin pretreatment, and was dependent on quercetin's ability to bind hemagglutinin proteins (HA). Specifically, quercetin bound (dose-dependently) the HA subunit responsible for membrane fusion during virus entry and virus-mediated hemolysis (69). *In vitro*, quercetin pre-treatment (10 μM) inhibited Rhinovirus (RV) virulence, entry, and replication into BEAS-2B cells via multiple mechanisms: it impeded RV endocytosis though misdirecting EEA1 localization -an early endosomal marker- and inhibiting AKT phosphorylation with subsequent 3-fold viral load reduction at 24 h, lowering negative-strand RNA and modulating interferon (IFN) and IL-8 expression (70). These results were confirmed *in vivo*, with an estimated lower plasmatic concentration of quercetin (nM) (similarly to other studies (71–73)) during which quercetin reduced RV-RNA at 1 day post-infection, modulated KC, MIP-2, TNF-a, and MCP-2, decreased virus-induced airway hyper-responsiveness, and modulated IFNs (IFN-α and IFN-λ2) (70).

### Interfering With DNA and RNA Polymerases

The *in vitro* antiviral effects of quercetin on herpesviruses (HSV-1, 2) and adenoviruses (ADV-3,−8,−11) suggest inhibition of early stage viral replication in a dose dependent manner (for HSV-1 100% inhibition at 60 mg/L) (16, 74), as well as inhibition of viral DNA and RNA polymerase (12, 75, 76). In human embryonic kidney cells (HEK), inoculated with polio, 3-methyl-quercetin disrupted plaque formation while quercetin itself demonstrated these effects when administered together with vitamin C (77). In fact, Vitamin C (either D- or L-ascorbate but not dehydroascorbate), prevented quercetin spontaneous degradation suggesting *necessary co-administration with ascorbate to exert its antiviral effect*. The beneficial effects of 3-methyl-quercetin (10 μM) were exerted primarily when the compound was administered 1–2 h post-poliovirus infection in Hela cells, inhibiting viral proteins and RNA synthesis in a dose dependent manner (78). In fact, 3-methyl-quercetin was identified as a molecule able to bind essential proteins required during the transcription from minus-strand RNA into positive polarity RNAs, thus interfering with cytoplasmic viral RNA replication (79).

In an *in vivo* study, a quercetin metabolite (4′,5-diacetyl-3,Y,7-trimethyl-quercetin), administered orally BID for 4 days protected mice against lethal infection by Coxsackie virus, promoting survival in a dose-response scale: 10, 20, and 40 mg/kg increased survival by 30, 40, and 50%, respectively (38). These beneficial effects were ascribed to a complete inhibition of virus replication when the compound was added within 2 h after virus absorption and related to the blockade of the RNA polymerase complex, as demonstrated *in vitro* (38).

### Inhibition of Reverse Transcriptase

Quercetin has been investigated *in vitro* as an antiviral agent for HIV due to its ability to inhibit crucial enzymes: reverse transcriptase (RT), integrase (IN), and protease (PR) (80). Quercetin significantly reduces HIV viral replication (81) and, when added to peripheral blood mononuclear cells (PBMNc) infected with HIV and compared to HIV infected controls, quercetin reduced the levels of p24, Long Terminal Repeat (LTR) gene expression, and viral infectivity together with an inhibition of TNF-α and upregulation of IL-13 (11).

Quercetin has also been shown to inhibit non-HIV RT activity *in vitro*, including avian myeloblastosis reverse transcriptase (AMV-RT), Rous-associated virus-2 (RAV-2-RT), and Maloney murine leukemia virus (MMLV-RT). Quercetin displayed a dose-dependent inhibitory action: at 50 μM, 23% inhibition of both AMV-RT and RAV-2-RT, and at 10 μM inhibition of mammalian MMLV-RT of almost 60% were reached (14). HIV-RT was inhibited completely at 2 μg/ml quercetin in a partially-competitive mode (76). These antiviral effects of quercetin are believed to be related to the five hydroxyl groups on 3, 3′, 4′, 5, and 7 as the inhibitory activity is lower for baicalein, quercetagetin, or luteolin which lack these groups (75).

Interestingly, Harakeh et al. studied the dose-dependent effect of ascorbic acid (0–150 μg/ml) on HIV-infected T-lymphocytes *in vitro* and reported that >99% reverse transcriptase and nearly >90% p24 antigen suppression and a 93% inhibition of syncytia formation, a marker that correlates with viral infectivity and cytopathic effects (82).

### Inhibition of Proteases

Quercetin is a potent HIV protease inhibitor *in vitro*, with an IC50 of 58.8 μM (83). Hepatitis C virus (HCV) NS3 serine protease catalytic activity was directly inhibited by quercetin treatment in a dose dependent manner (95% NS3 inhibition at 1.25 mg/ml); in this study quercetin blocked virus RNA production and impeded virus replication by 70% at 72 h without affecting cell viability (13).

### Blocking Virus Assembly

Quercetin treatment inhibits HCV replication (84). This effect is attributed to its ability to modulate Heat Shock Protein expression (HSPs), thus impeding the crucial binding between heat shock factor and elements (HSF-HSE) necessary for the stress-induced transcription of stress genes (85, 86). Quercetin reduced HSP70 and HSP40, thereby impeding the formation of Non-Structural protein 5A complexes (NS5A-HSP70 and NS5A-HSP40) necessary for HCV genome replication apparatus through the internal ribosome entry site (IRES). Despite unaltered HCV titer, the production of infectious particles was decreased, interestingly more by quercetin treatment than by HSP knockdown, displaying a dose-dependent relationship: at 0.5 μM quercetin reduced viral production by 29%, at 5 μM by 90%, and at 50 μM by nearly 100% (84).

### Immunomodulatory Properties

Quercetin stimulates T-helper cells to produce (Th-1)-derived Interferon-γ (IFN- γ) and downregulates Th2-derived IL-4 when added to cultured blood peripheral mononuclear cells (11). Immunonutrition studies in mice with supplementary polyphenols, including quercetin, showed enhanced NK cell lytic activity, neutrophil chemotaxis, and lymphocyte proliferation (87, 88).

Human foreskin fibroblast (HFF) and endothelial cells (EC) pretreated with 2-phospho-ascorbate (ASC-2P) resisted CMV infection; they displayed a reduction in immediate and late antigens and viral yield was inhibited 50–100-fold in ECs and 100–1,000-fold in HFF (89). This effect was not dependent on a sustained ASC-2P presence but was abolished if the ASC2-P was added after the virus infection, indicating an immunomodulatory effect, rather than directly antiviral. Animal models with gulo (–/–) mice insufficient in vitamin C, when infected with 20 hemagglutination units (HAU) of H3N2 influenza exhibited worse outcomes than wild type and Gulo (–/–) sufficient in vitamin C (90). Gulo (–/–) showed a reduction in IFN-α/β while displaying higher levels of IL-1α, TNF-α, and IL-1B. When Gulo (–/–) mice received supplemental Vitamin C, these cytokine expression profiles were lost.

Patients with acute Epstein-Barr infection (EBV) treated with high doses of intravenous vitamin C (7.5–50 g) displayed lower EBV-IgG (levels, while EBV VCA IgM antibody levels were negatively correlated to increasing plasma ascorbate concentration (91). Patients with HTLV-1-associated myelopathy/tropical spastic paraparesis HAM/TSP were all successfully treated with 35–40 mg/kg oral vitamin C for 3–5 days despite no changes in serum HTLV-1 or CSF HTLV-1 antibody titer, indicating an immunomodulative effect (92). Of these patients, 4 underwent a vitamin C on-off study which demonstrated a “*positive dose response relationship with neurological symptoms*.” A separate prospective trial into a diverse number of therapies indicated that vitamin C improved motor disability grades in HAM/TSP in 20% of patients (93). High dose ascorbic acid was then shown to display antiproliferative (95% decrease in lymphoproliferation) and immunomodulatory effects (via reduction of TNF-α, IFN-γ, IL-6, and p19) in peripheral blood mononuclear cells (PBMCs) extracted from HAM+ patients and T helper cell lines.

Vitamin C administration has been related to enhanced interferon production and was studied for its possible use for the prevention of vaccine failure. Rabies vaccination, when supplemented with 2 g oral vitamin C for each of the 3 injections provoked, at 24 h, increased serum IFN-α levels, indicating that “vitamin C is an effective stimulator of interferon production” (94). Mice on an *ad libitum* diet containing vitamin C increased induction of interferon (62–145%) depending on the viral titer of inoculation (95), and L-ascorbate added to stimulated mouse cell lines increases interferon synthesis (96). Low levels of vitamin C, in fact, have been related to insufficient phosphorylation of signal transducers and activation of transcription (STATs), which represent a crucial signaling process of IFNs (97). Specifically, T cells of mice deficient in vitamin C display defects in STAT3 phosphorylation (90).

## Focus on SARS-CoV-2

Quercetin has been investigated for its possible antiviral effect on several members of the *Coronaviridae* family and, as mentioned by Ling Yi and colleagues, “*quercetin offers great promise as a potential drug in the clinical treatment of SARS*” (98). SARS-Coronavirus, described in 2003 (99), is a single-stranded RNA virus of ~29,700 nucleotides, which uses ribosome sites to encode two replicase glycoproteins, PP1a and PP1b, that mediate viral replication (99, 100). Once these precursor glycoproteins are synthesized, 3C-like protease (3CLpro) plays a critical role in the lytic release of its replicates (101). Quercetin-3β-galactoside binds SARS-Cov 3CL protease and inhibits its proteolytic activity with an IC50 of 42.79 ± 4.95 μM (102). This inhibitory action on 3CLpro is dependent on the hydroxyl group of quercetin which, as shown through molecular modeling and Q189A mutation, recognizes Gln189 as a crucial site on 3CLpro responsible for the binding of quercetin (102). Quercetin was also identified as a compound able to block SARS-Coronavirus entry into Vero E6 cells with a half-effective concentration (EC50) of 83.4 μM and with low cytotoxicity (CC50 3.32 mM) (98).

SARS-CoV-2, the virus responsible for the 2020 COVID-19 pandemic (103), belongs to the genus *Betacoronavirus* and subgenus *Sarbecovirus* and, due to its similar receptor-binding domain, it is assumed, similarly to SARS-CoV, to infect type II pneumocytes entering via the angiotensin-converting enzyme II receptor (104). SARS-Cov-2 protease 3CL maintains the same Gln189 site (105) of SARS-Cov 3CLpro, which previously was identified as the binding site for the hydroxyl groups of quercetin and its derivates (102).

Interestingly, an *in vitro* study of ascorbic acid treatment on chick-embryo ciliated tracheal organ cells (CETO) promoted resistance to Coronavirus infection but did not show any effect on orthomyxovirus or paramyxovirus (106).

Despite the breadth and depth of anti-viral *in vitro* and *in vivo* studies into the immunomodulatory effects of quercetin and vitamin C administration, further studies are absolutely necessary to confirm quercetin inhibitory activities on SARS-Cov-2 virus entry, RNA polymerase, and on other necessary viral life-cycle enzymes.

## Pharmacokinetics of Quercetin

Orally administered quercetin glycosides are hydroxylated by β-glucosidases in the gut (107, 108). Aglycone quercetin passively permeate the intestinal epithelial barrier while quercetin glycosides are absorbed via the intestinal sodium/glucose cotransporter-1 (109). The bioavailability of oral quercetin is extremely variable, achieving values from 0 to 50% (110). Quercetin can be metabolized either in the enterocytes or in the hepatocytes forming glucuronidated, sulfated, or/and methylated compounds (111). Indeed, four out of five hydroxyl groups of quercetin can be glucuronidated by UDP-glucuronosyltransferase, forming its major metabolites: quercetin-3-glucuronide, 3'-methylquercetin-3-glucuronide, and quercetin-3'-sulfate (112). Rat tissue distribution of orally, long-term administered quercetin (12 weeks) shows the highest concentration in the lungs while pigs display the highest concentrations in the liver and kidneys (113). In contrast, short-term administration exhibits no marked distribution, implying that the beneficial effects of quercetin in preventing lung respiratory viral infection could be maximized by long-term administration. Following 500 mg oral quercetin, maximum plasma concentration of ~15 μg/L of aglycone quercetin (~50 nM, T_max_ of 3 h) and 450 μg/L of quercetin non-methylated conjugates (T_max_ of 4 h) were found (114). Intravenous administration results in an elimination half-life of 0.7–2.4 h with a distribution volume at steady-state of 6.2 to 92.6 L and with a total body clearance of 30 h (110).

## Safe Profile and Optimal Dosing

Oral supplementation with quercetin up to 1 g/day for 3 months has not resulted in significant adverse effects (111). In a randomized placebo-controlled study, 30 patients with chronic prostatitis were supplemented with oral quercetin (1 g/day) and reported only two mild adverse reactions (headache and temporary peripheral paresthesia) (115). Intravenous administration of quercetin in a phase I clinical trial for cancer patients resulted in nausea, vomiting, sweating, flushing, and dyspnea at doses >10.5 mg/Kg (756 mg per 70 Kg individual) (116). Only higher intravenously administered doses up to 51.3 mg/Kg (around 3,591 mg per individual) were associated with renal toxicity (111). The safety of quercetin-based oral supplementation during pregnancy and breastfeeding has not been established.

We have previously described the safety profile and dosing strategies of vitamin C (117). According to the data presented above, we propose the following optimal dosing (Table 1). Further studies are needed to examine and discuss the possible administration of quercetin for prolonged periods of time (>1 year).

**Table 1:** Proposed multi-drug approach for either the prophylaxis for high risk population, and treatment of mild and severe cases.

	**Quercetin**	**Vitamin C**
Prophylaxis	250–500 mg BID	500 mg BID
Mild cases	250–500 mg BID	500 mg BID
Severe Cases^*^	500 mg BID	3 gr q6 for 7 days

* *ARDS-like presentation, require assisted ventilation/intubation, ICU hospitalization*.

## Synergistic Antiviral Action

**Figure 2:**
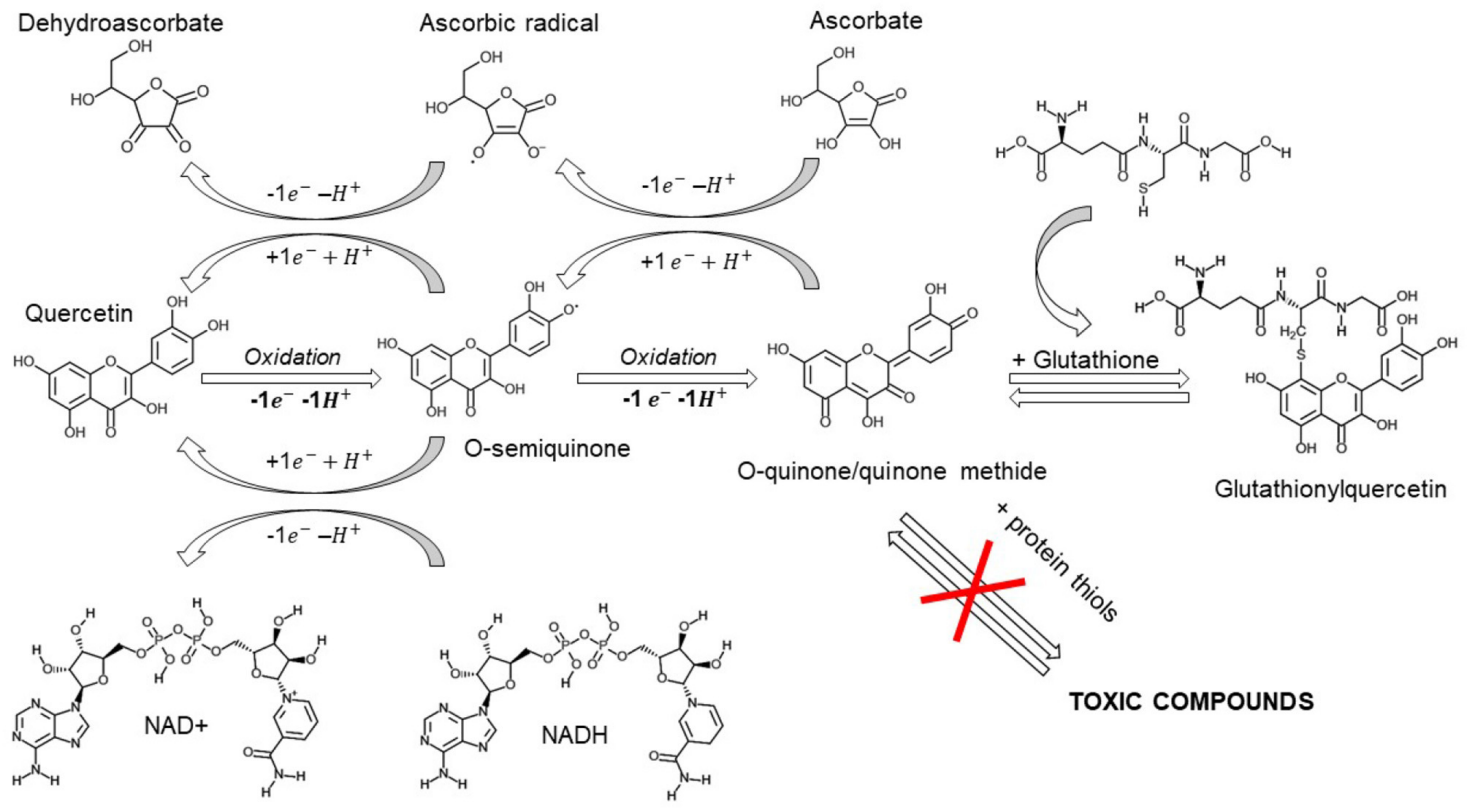
After exerting its scavenging properties, quercetin is oxidized into its reactive products o-semiquinone and o-quinone/quinone methide (QQ). These compounds can be recycled by antioxidants like ascorbate or NADH or removed by glutathione. If ascorbate or glutathione levels are reduced, QQ can bind protein thiols producing transient toxic compounds. Created with ChemDoodle Web with permission (18).

Quercetin spontaneously oxidizes to form O-semiquinone and O-quinone/quinone methide (QQ), which can bind protein thiols forming toxic compounds (118). This process of both anti- and pro-oxidant effects has been named the “*quercetin paradox*” (119). However, QQ can be recycled into quercetin by electron donors like NADH or ascorbate, or form together, with glutathione either 6-glutathionyl-quercetin or 8-glutathionyl-quercetin (GSQs) (107, 120). Importantly, if ascorbate or glutathione levels are *insufficient*, quercetin may be shunted to QQ and exert prooxidant effects. Therefore, we stress the importance for its co-administration with vitamin C (121, 122). However, even though QQ exhibits a higher affinity for glutathione than for vitamin C (121), the methylated metabolites of quercetin show a higher preference for ascorbate than for thiols, suggesting a cycling of activity which will exert anti-oxidant effects (Figure 2) (123). Furthermore, both GSQs (124) and QQ-protein thiols have been shown to be unstable and transient -lasting for minutes and hours instead of days- suggesting an overestimation of the proposed *in vitro* toxicity (125).

The supraphysiological concentrations of ascorbate achieved with intravenous administration (i.v. 3 gr q6) are capable of free radical scavenging and electron donation, preventing either quercetin or glutathione oxidation. In this scenario, ascorbate may exert antioxidant and immunoprotective effects, quercetin and its metabolites exert a concurrent antiviral response and, if quercetin-oxidized compounds are formed, they can be partially recycled by ascorbate and transported by glutathione, thus preventing their possible toxicity.

## Discussion

A multi-drug approach with quercetin and vitamin C may disrupt virus entry, replication, enzyme activity and assembly, and concurrently fortify the immune response promoting early IFNs production, modulating interleukins, promoting T cell maturation, and phagocytic activity. Quercetin and ascorbic acid co-administration represents an experimental strategy for prophylaxis and treatment of several respiratory viruses, such as SARS-CoV-2. The blockage of virus entry represents a key strategy and quercetin impedes viral membrane fusion for both influenza and SARS-Cov *in vitro* (98). Quercetin also targets viral polymerases and may disrupt replication *via* the inhibition of reverse transcriptase enzymes. Quercetin further inhibits SARS 3CL protease by binding to its GLN189 site (102), which is expressed similarly by SARS-COV-2 (105) and provides a direct mechanistic rationale for its experimental clinical use—in addition to its immunoenhancing and anti-inflammatory actions. Despite the limitations of *in vitro* research, it is noteworthy that the few *in vivo* models reviewed here indicate increased survival from lethal viral infection when treated with quercetin (42, 64). Some studies suggest that oral administration and metabolic processing (methylation, conjugation, etc.) is necessary, and have identified quercetin derivates, which display variable T_max_, as responsible for a cooperative antiviral activity (126–128).

Vitamin C exerts immunomodulatory activity, enhancing interferon production through STAT3 phosphorylation (90), limiting cytokine-induced organ damage (55), promoting survival in lethal infections (54) and, importantly, is able to recycle oxidized quercetin (120), enhancing its antiviral effects. SARS-Cov-2 virus infection may initiate a strong inflammatory and dysregulated reaction in the lung with increased levels of IL-6 and a “cytokine-storm” (129) which has been shown to provoke either an asymptomatic, mild, or severe infections This cytokine dysregulation may be associated with neutrophil extracellular traps (130) and alterations in T cell activity (131). These immunological alterations which have characterized our current understanding of Covid-19 suggest that agents which target immune modulation, rather than direct viricidal activity, may present exciting targets for pharmacological intervention. In this scenario, Vitamin C and quercetin co-administration may represent a safe, effective, and inexpensive antiviral and immunomodulative approach for both the prophylaxis of high-risk populations and the treatment of both mild and severe cases.

They have also consistently been shown to display excellent safety profiles, and a consideration of risks and benefits in their therapeutic potential should be placed within this context. Vitamin C is a widely available supplement which many millions of people use already, and we have highlighted its antiviral properties in conjunction with quercetin. Due to its large-scale use, vitamin C in particular would be a cheap intervention with which to ascertain these compounds' efficacy as a prophylactic intervention. The prophylactic use of over-the-counter vitamin supplementation to combat infection is a behavior many people engage with already. Research into the potential prophylactic administration of vitamin C and quercetin in high-risk groups is therefore warranted.

The excellent side effect profile of these agents would also suggest that they may complement interventions which have displayed potential benefits in treating Covid-19, such as Remdesivir (132) and convalescent plasma (133, 134), which we believe warrants their experimental use in clinical trials.

There are potential limitations of their use in clinical studies. Both agents are present in varying degrees in individuals' diets and global recommendations for vitamin C intake vary extensively across the globe (135). Prophylactic interventions in general populations within the community will therefore be confounded by the quantity present in differing diets. Agents such as vitamin C also have well-characterized beneficial effects apart from the antiviral properties we have highlighted here. Supplementation with these agents may therefore promote general health and indirectly affect the capacity of individuals to combat viral infection. Although this would diminish the ability to identify the direct antiviral properties of vitamin C in clinical studies it may have ancillary benefits of promoting general health, which may be particularly pertinent if administered in communities with greater deprivation or from less economically developed countries.

## Conclusion

Quercetin displays a broad range of antiviral properties which can interfere at multiple steps of pathogen virulence -virus entry, virus replication, protein assembly- and that these therapeutic effects can be augmented by the co-administration of vitamin C. Furthermore, due to their lack of severe side effects and low-costs, we strongly suggest the combined administration of these two compounds for both the prophylaxis and the early treatment of respiratory tract infections, especially including COVID-19 patients.

## Author Contributions

All authors listed have made a substantial, direct and intellectual contribution to the work, and approved it for publication.

## Conflict of Interest

The authors declare that the research was conducted in the absence of any commercial or financial relationships that could be construed as a potential conflict of interest.
